# Thermal adaptation of pelage in desert rodents balances cooling and insulation

**DOI:** 10.1111/evo.14643

**Published:** 2022-10-18

**Authors:** Eric A. Riddell, James L. Patton, Steven R. Beissinger

**Affiliations:** ^1^ Museum of Vertebrate Zoology University of California, Berkeley Berkeley California 94720; ^2^ Department of Ecology, Evolution, and Organismal Biology Iowa State University Ames Iowa 50010; ^3^ Department of Environmental Science, Policy, and Management University of California, Berkeley Berkeley California 94720

**Keywords:** Adaptation, climate change, mammals, morphological evolution, temperature

## Abstract

Phenotypic convergence across distantly related taxa can be driven by similar selective pressures from the environment or intrinsic constraints. The roles of these processes on physiological strategies, such as homeothermy, are poorly understood. We studied the evolution of thermal properties of mammalian pelage in a diverse community of rodents inhabiting the Mojave Desert, USA. We used a heat flux device to measure the thermal insulation of museum specimens and determined whether thermal properties were associated with habitat preferences while assessing phylogenetic dependence. Species that prefer arid habitats exhibited lower conductivity and thinner pelage relative to species with other habitat preferences. Despite being thinner, the pelage of arid species exhibited comparable insulation to the pelage of the other species due to its lower conductivity. Thus, arid species have insulative pelage while simultaneously benefitting from thin pelage that promotes convective cooling. We found no evidence of intrinsic constraints or phylogenetic dependence, indicating pelage readily evolves to environmental pressures. Thermoregulatory simulations demonstrated that arid specialists reduced energetic costs required for homeothermy by 14.5% by evolving lower conductivity, providing support for adaptive evolution of pelage. Our study indicates that selection for lower energetic requirements of homeothermy has shaped evolution of pelage thermal properties.

Phenotypic similarity across distantly related taxa—often referred to as convergent evolution or homoplasy—can be driven by natural selection from environmental pressures or intrinsic constraints (Losos 2011; Wake et al. 2011). Evidence suggests natural selection can shape phenotypic convergence by favoring similar strategies to cope with the same selective pressures in different environments (Losos 2011). However, intrinsic constraints, such as physical limitations or developmental processes, might also result in the repeated evolution of similar strategies (Maynard Smith et al. 1985). Both adaptation to the environment and internal constraints have been shown to drive the convergence of morphological variation (Losos 1990; Grizante et al. 2010; Stoessel et al. 2013; Stepanova and Womack 2020). Few studies have examined the processes driving convergence in traits related to physiological strategies, such as homeothermy in endotherms (Angilletta et al. 2010).

Natural selection is likely to shape traits that influence homeothermy due to the important consequences of energy balance for reproduction, survival, and performance (Burton et al. 2011). However, selection could target a variety of morphological, physiological, and behavioral traits involved in endotherm thermoregulation (Martinez et al. 2020), making the exact target of selection unclear. Moreover, providing support for the adaptive benefits of these traits for homeothermy is often lacking due to the complex biophysical processes affecting heat flux in natural environments (Bakken 1981). Simulations of heat flux, therefore, can evaluate the role of environmental pressure in driving selection on phenotypic convergence (Losos 2011) and reveal insight into the basis of adaptive evolution.

Mammal pelage readily evolves in response to a variety of selective pressures in the environment, especially the thermal environment. The thermal environment imposes selective pressure on endotherms because they tend to maintain a relatively stable body temperature across a wide range of environmental temperatures, particularly when active (Ruben 1995). The insulation provided by mammalian pelage influences the rate at which heat is transferred between mammals and their environment and, as a result, the physiological requirements for metabolic heat production and evaporative cooling for thermoregulation (Bakken 1976, 1991; Conley and Porter 1986; Geist 1987; Steudel et al. 1994). Studies of the thermal properties of pelage have historically focused on the benefits of pelage in cold environments (Morrison and Tietz 1957; Chappell 1980; Russell and Tumlison 1996; Scholander et al. 1950a, 1950b), dramatic seasonal changes in pelage (Jofré and Caviedes‐Vidal 2003; Boyles and Bakken 2007), or the potential for pelage to influence body size clines of endotherms (Geist 1987; Steudel et al. 1994; Briscoe et al. 2015). To live in cold environments, mammals have evolved longer and denser pelage, which promotes heat retention (Walsberg 1991). However, less is known about the evolution of pelage in hot environments. Some desert mammals adjust the solar reflectance of pelage to absorb more heat in colder months (Walsberg and Schmidt 1989; Walsberg et al. 1997). Desert rodents may also possess less dense pelage, resulting in greater capacity for convective cooling by wind (Goodfriend et al. 1991). Pelage can also be stiff, providing greater resistance to convective heat transfer by maintaining the layer of air next to the skin (Russell and Tumlison 1996). In desert ungulates, larger species have thinner pelage that conducts less heat relative to small species (Hofmeyr 1985). Nevertheless, we lack comparative studies of the thermal properties of pelage that tease apart the underlying processes driving phenotypic convergence.

We compared the thermal insulation and conductivity of pelage among a community of rodents that inhabit the Mojave Desert, USA to understand the evolutionary processes driving convergence in pelage. Here, we define pelage as the fur of a specimen (often referred to as “flat skin”) and focus on the thermal properties of the fur (rather than the skin). We measured insulation on museum specimens using a heat flux device designed to measure the rate at which heat is transferred across the pelage, known as insulation or conductance (with insulation being the inverse of conductance). We also calculated conductivity to understand how heat travels through the pelage per unit thickness of the pelage. We then used a phylogenetic approach to evaluate the degree to which relatedness among species contributed to thermal properties of pelage while also assessing morphological and ecological traits, such as habitat preferences. Finally, we used heat flux simulations to evaluate the potential adaptive benefits of the thermal properties of pelage and assessed the degree of intraspecific variation to evaluate potential intrinsic constraints (Losos 2011).

We predicted that species inhabiting cooler, less arid habitats (e.g., woodlands) would exhibit higher insulation compared to species that prefer hot, more arid habitats. We also predicted that the observed differences in insulation would be driven by longer, deeper pelage, which acts to trap more air for insulation (Hammel 1955; Hart 1956). Pelage properties are also likely to vary by body size because longer pelage impedes locomotion in smaller species (Wasserman and Nash 1979; Steudel et al. 1994). As a result, conductivity of pelage should be lower in smaller rodents than in larger rodents if selection favors greater heat retention in small‐bodied endotherms. We present the first study to evaluate the evolution of thermal properties of pelage in a comparative framework to identify adaptations related to living in hot, arid environments.

## Methods

### HEAT FLUX MEASUREMENTS

We measured the thermal insulation of pelage from specimens curated in the Museum of Vertebrate Zoology (MVZ) at the University of California, Berkeley. We selected 163 flat and intact skin specimens (Table S1) representing 25 of the 34 species that inhabit the Mojave Desert, USA (Riddell et al. 2021). Each species exhibits fossorial behavior by using underground burrows for shelter, and patterns of aboveground activity are often sensitive to the thermal environment (Chappell and Bartholomew 1981). Each specimen was air dried without chemical preservation and maintained in standard museum cases. All subcutaneous tissue from the specimen was removed.

Although each of the 25 species we measured occur within the Mojave Desert, the available museum specimens included specimens from all three North American deserts: the Great Basin (38.2%), Mojave Desert (30.1%), and Sonoran Desert (29.3%). Two additional specimens (1.6%) were collected elsewhere. These desert regions are characterized by extreme temperatures (regularly exceeding 40°C in the summer and −10°C in the winter) and little annual precipitation (<400 mm), which mostly comes during winter (Beatley 1974; Urban et al. 2009). The landscape is dominated by creosote and bursage, which provide 20% shade cover on average (Urban et al. 2009). Most (61.3%) specimens were collected in warmer months (April to October) and the remainder (38.7%) in cooler months (November to March). All specimens were adults and attempted to achieve equal representation of males and females for each species. However, only a single flat skin specimen was available for five species (*Dipodomys ordii*, *Neotamias merriami*, *Neotamias panamintinus*, *Microtus longicaudus*, *Peromyscus boylii*). All field procedures for modern collections followed the guidelines of the American Society of Mammalogy (Sikes et al. 2011).

Measurements of thermal conductance are typically taken on freshly prepared specimens and often require destruction of the specimen. We developed our technique to nondestructively measure the thermal conductance of museum specimens, while also assessing the potential effects of making measurements on dried specimens collected over the last century. We built a custom device to measure the thermal conductance of mammal pelage (Fig. S1). Our approach was inspired by similar techniques that measured temperature gradients and heat flux across pelage in a controlled laboratory setting (Cena and Monteith 1975; Walsberg et al. 1978). Our device consisted of a copper pipe (2.54 cm in diameter) that circulated warm water controlled at 37°C to simulate the normothermic body temperature of small mammals. We pumped water using a submersible water pump (Aquatop, N‐302) from a temperature‐controlled bath maintained at 37°C into the copper pipe. The copper pipe was embedded into a 6‐inch cube of polystyrene to minimize heat transfer away from the pipe. The tip of the copper pipe was flush with the foam insulation to direct the flow of heat from the warm copper pipe into the flat mammal skin positioned on top of the copper pipe. The entire apparatus was placed inside a temperature‐controlled incubator chamber (ReptiPro 6000) held at 30°C.

We measured the temperature gradient across the mammal pelage using a heat flux sensor (FluxTeq, PFHS‐01) and a precision wire type‐T probe thermocouple (Thermoworks, PT‐6). The heat flux sensor (surface area = 1.6 cm^2^) was a differential‐temperature thermophile made of Kapton (polyimide) with a sensor thickness of approximately 305 microns. The heat flux sensor contains a type‐T thermocouple embedded into the sensor to simultaneously measure heat flux (W/m^2^) and temperature (°C) across the sensor surface. The sensor was calibrated using a conduction‐based system by placing the sensor between a precisely controlled hot plate surface warmed with a resistance heater and a cold water‐cooled surface (conducted by manufacturer). The calibration procedure developed by FluxTeq LLC (www.fluxteq.com) provides estimates of heat flux with 95% accuracy.

The heat flux sensor was placed on top of the copper pipe and secured using double‐sided tape, following FluxTeq LLC protocols. Then, we applied a thin layer of petroleum jelly (Vaseline^TM^) to the skin on the underside of the specimen to ensure that the skin was sealed to the heat flux sensor without any air pockets (Walsberg et al. 1978). After the flat mammal specimen was placed on top of the copper pipe, we adhered the specimen to the heat flux sensor using a clamping system that pressed the specimen between the polystyrene insulation and wooden surface. We measured a single estimate of heat flux across the dorsal midline of each specimen, with each measurement taking roughly 30–45 m to come to thermal equilibrium. We drilled a hole (1.6 cm^2^) in the middle of the wooden surface that aligned with the copper pipe apparatus, and the hole was lined with a clear vinyl rubber tube to direct the heat flow through the pelage for measurement. The platform ensured the specimen laid flat on the heat flux sensor while allowing measurement of heat flux without flattening or affecting the pelage (Cena and Monteith 1975; Walsberg et al. 1978). We measured heat flux in the middle of the ring using a thermocouple (Fig. S1). We secured the entire device using DeWalt^®^ Trigger Clamps to adhere the museum specimen tightly to the copper pipe.

We recorded the heat flux and temperature from various thermocouples using an analog voltage measurement system (FluxTeq, FluxDAQ) with an integrated thermistor for cold junction temperature compensation. The FluxDAQ continuously measured the temperature of the copper pipe, heat flux across the copper pipe into the skin, temperature above the pelage, air temperature, and wall temperature of the incubator. We used air temperature and wall temperature measurements to ensure black‐body conditions within the chamber (Walsberg et al. 1978). We measured the temperature gradient across the pelage by calculating the difference between the temperature of the heat flux sensor and at the tips of the hair. We secured the type‐T bead probe (gauge = 0.07366 cm) at the tips of the hair using an alligator clip. We then calculated thermal conductivity as

$$H=-k\left(\frac{dT}{dx}\right),$$

where *H* is the heat flux from the heat flux sensor (W/°C), *k* is the thermal conductivity, *T* is the temperature gradient (°C), and *x* is the thickness of the pelage (m) (Walsberg 1988). After the readings had come to equilibrium, we recorded the average conductance value over 90 s. We were unable to measure heat flux across the ventral portion of the pelage, which likely exhibits different thermal characteristics than the dorsal pelage (Conley and Porter 1986), because ventral portions were generally too small to measure accurately using our heat flux device. Similar studies have also experienced the same problem (Boyles and Bakken 2007).

We also compared the thermal insulation of freshly prepared skins and dried skins to assess the consequences of using museum specimens for thermal measurements. First, we measured thermal conductance of freshly prepared skins (*n* = 5) from a colony of *Mus musculus* maintained by the Nachman Lab at the MVZ. We then air dried the specimens for at least 2 weeks, which mirrored the same protocol as the measured museum specimens. We measured the thermal conductance using the methods described above. Pelage conductance of fresh and dried specimens met the assumptions of normality (Shapiro‐Wilk test: *P* = 0.77). The mean conductance of freshly prepared skins was 15.3% (standard error = 1.2%) higher compared to the dried specimen (paired *t*‐test: *p* < 0.001). We used the correction factor to compare thermal properties of rodent pelage from this study to previously published data from fresh mammal skins (Scholander et al. 1950c), which we collected using DataThief III (version 1.7). Pelage insulation from our study corresponded closely to the expected relationship between insulation and pelage thickness (Fig. S2). We acknowledge these correction values may differ by species, but species‐specific correction values are unlikely to alter our conclusions based on the consistency of our measurements with previously collected values (Fig. S2). Regardless, we elected to use the uncorrected values in our analyses. The use of freshly prepared specimens was approved by the University of California, Berkeley Institutional Animal Care and Use Committee (AUP‐2017‐08‐10248).

After the conductance measurements, we recorded length of the hairs and thickness of the pelage from museum specimens, where we define length as the distance from the outer surface of the skin to the tip of the hair and thickness as the vertical distance from the outer surface of the skin to the outer surface of the hair. Prior to taking measurements, we ensured that the pelage was not laying irregularly. We measured the length of hairs at three locations spanning the dorsal side of the specimen at the approximate locations of the thoracic, lumbar, and sacral vertebrae. We placed the base of a Fisherbrand^TM^ 150 mm ruler at the base of the hair and pressed the hair against the ruler to measure the length to the nearest 0.5 mm. We also measured thickness at the same three locations spanning the dorsal side of the specimen. The density of the pelage (hairs per unit area) can play an important role in its thermal properties (Tregear 1965; Wasserman and Nash 1979), but we were unable to sample it due to the labor‐intensive process. Thus, the role of density on the thermal properties of pelage remains unknown in our study. Nevertheless, any differences associated with pelage thickness are likely a combination of density and thickness because they are often correlated (Tregear 1965).

### PHYLOGENY

We used a recently developed database (PHYLACINE, version 1.2) consisting of phylogenies with 5831 known mammal species built with a hierarchical Bayesian approach (Faurby and Svenning 2015; Faurby et al. 2018). We selected the smaller phylogenetic tree consisting only of species with genetic data and relationships in which topological placement was unambiguous, because the tree contained all of our species of interest. Genetic data for the phylogeny originated from studies encompassing nearly all mammalian families, molecular phylogenies with more than one independent marker, and genus‐level phylogenies based on a single marker (Meredith et al. 2011; Faurby et al. 2018). We downloaded the full set of trees (*n* = 1000) to assess phylogenetic uncertainty in our analyses and trimmed the mammal phylogeny to the 25 desert rodents in our study. For illustration purposes, we mapped thermally relevant traits onto an averaged phylogenetic tree using consensus approach in *phytools*.

### HEAT FLUX SIMULATIONS

To evaluate support for adaptive evolution of thermal conductivity of pelage, we used heat flux simulations (Endoscape; www.github.com/ecophysiology/Endoscape) designed and validated for this community of small mammals in the Mojave Desert (see Riddell et al. 2021 for a detailed explanation of the model and validation). These simulations use biophysical principles to estimate the rate at which mammals lose or gain heat to their environment based upon

$$Q=M-E-C\frac{dT_{b}}{dt}=K_{e}\left(T_{b}-T_{e}\right),$$

where *Q* is the net sensible heat flux, *M* is the heat generated through metabolic processes, *E* is the heat lost via evaporative processes, *C* is the heat capacitance of the isothermal core, *T*_b_ is body temperature, *t* is time, *K*_e_ is the effective conductance, and *T*_e_ is the operative temperature (Bakken 1981). By estimating rates of heat flux, the model calculates the amount of energy that would need to be generated via metabolic heat production (i.e., thermoregulatory heating costs) or lost via evaporative cooling (i.e., thermoregulatory cooling costs) in complex thermal environments. We estimated heat flux for both nocturnal and diurnal species (i.e., nocturnal individuals ceased activity on the surface when the sun was above the horizon). While inactive, individuals retreated to underground burrows based on the soil depth associated with the geographic location of the simulation (range: 0.13–4 m).

To assess variation in thermoregulatory costs, we ran the simulations for 90 sites across the Mojave Desert assuming average climatic conditions for each month of the year (Riddell et al. 2021). We used *NicheMapR* to generate microclimatic conditions for each site under contemporary climates assuming 50% shade cover to incorporate the effects of vegetation (Flint et al. 2013; Kearney and Porter 2020). We assume that desert rodents remain active throughout the year (i.e., no periods of dormancy or hibernation); however, each species often exhibits unique patterns of activity in different seasons (Reid 2006). Therefore, we also report thermoregulatory costs for each month of the year to understand how pelage conductivity influences metabolic heat production in different seasons. In a supplemental analysis, we also incorporated torpor by assuming body temperatures fell to 17°C during inactivity underground based upon the average value associated with daily bouts of torpor for mammals (Ruf and Geiser 2015). Although desert rodents exhibit species‐specific minimum body temperatures during torpor (Ruf and Geiser 2015), the purpose of this supplementary analysis was to evaluate the sensitivity of our conclusions to torpor—not to generate species‐specific estimates of thermoregulatory costs.

We ran two scenarios using our heat flux simulations to determine how the evolution of lower pelage conductivity influenced heat loss to the environment. In the first scenario, we ran simulations using the average conductivity of pelage for arid specialists (0.0353 W/m/K). In a second, hypothetical scenario, we used the average conductivity across generalists, grassland species, and woodland species (0.0503 W/m/K). These values were determined from the model estimates for each habitat preference. We also ran supplementary analyses assuming a 15% reduction in the conductivity of pelage (as discovered by freshly compared specimens above). In the simulation, the conductivity of the skin also changed dynamically depending upon environmental conditions to simulate vasodilation and vasoconstriction (Riddell et al. 2021). Most species in our dataset were nocturnal (84% or 21/25), with the exception of *N. panamintinus*, *N. merriami*, *Ammospermophilus leucurus*, and *Otospermophilus beecheyi*. We ran each analysis for a nocturnal and diurnal species using the same physical parameters. We held all other traits constant based on the average values for arid specialists, including body mass, body shape, pelage depth, pelage length, and emissivity (see Table S2 for parameters). Therefore, these simulations were designed to evaluate the thermoregulatory consequences for the evolution of lower thermal conductivity in arid specialists. Given the strong potential for selection against high metabolic costs (Burton et al. 2011), lower metabolic heat production provided by lower thermal conductivity would provide support for natural selection driving convergent evolution of pelage conductivity.

### STATISTICAL ANALYSES

All statistical analyses were conducted in *R* (version 3.4.2, “Short summer”). We conducted phylogenetic least squares analyses (PGLS) to account for nonindependence of species phenotypes due to shared ancestry (Blomberg et al. 2003). We used the *pgls*() function in the *caper* package to evaluate whether species traits and pelage properties were associated with the thermal conductivity and insulation of pelage while simultaneously calculating Pagel's *λ* to assess phylogenetic signal. For the PGLS, we generated a single consensus phylogeny across the 1000 trees using the *ls.consensus()* function from *phytools* (Revell 2012) and *midpoint*() from *phangorn* (Schliep 2011) (see below for treatment of phylogenetic uncertainty). For each species, we assigned habitat preferences and activity type based upon field guides (Reid 2006) and mass (ranging from 7.6 to 578.5 g) from the PHYLACINE database (Faurby and Svenning 2015; Faurby et al. 2018). We conducted separate analyses on conductivity and insulation. For conductivity, we assessed the influence of log‐scaled body mass, length of the dorsal hairs, activity type (diurnal or nocturnal), and habitat preference (desert scrub, woodland, grassland, and generalist). For insulation, we used the same covariates and also included thickness of the pelage. Thickness was not included in the analysis on conductivity because it is used to calculate conductivity (see equation above). Model covariates were scaled and centered to compare slope estimates.

We assessed collinearity using a variance inflation factor (GVIF^1/(2*df)^), which indicates problematic collinearity if values are greater than 2 (Fox and Monette 1992; Zuur et al. 2010). We did not eliminate variables because variance inflation factors were less than two for each parameter. To assess variable significance, we used the *procD.pgls*() function from the *geomorph* package (Adams and Otárola‐Castillo 2013). We conducted a Type II analysis of covariance (ANCOVA) because it is preferred for unbalanced data (Langsrud 2003). We provided *Z* statistics to indicate effect size (Adams and Otárola‐Castillo 2013). To accommodate potential phylogenetic uncertainty in our tree, we ran the conductivity and insulation models for all 1000 trimmed phylogenies. We report the median value of Pagel's *λ* and median *P*‐values associated with determining whether the statistic was different from zero or one. We then compared the median phylogenetic signal with the phylogenetic signal quantified using the median consensus phylogenetic tree. By comparing the results from the consensus tree and across all phylogenies, we evaluated the degree to which the consensus tree (and resulting parameter estimates for the PGLS) was sensitive to phylogenetic uncertainty.

We also analyzed the conductivity and insulation models using nonphylogenetically informed ANCOVA. For this analysis, we used the full dataset consisting of 163 measurements (rather than species averages used in the PGLS) to assess the consistency of our results by including intraspecific variation. Statistics were calculated from scaled and centered covariates, but for plotting relationships, we used uncorrected values to increase interpretability. Out of the 163 specimens, 12 were missing mass data (7.4%); thus, we used the species‐specific mass estimates from THYLACINE (see above). We calculated the effect size (*ω* ^2^) of predictors to evaluate the relative influence of variables on insulation and conductivity (Olejnik and Algina 2003). Using the full dataset, we also evaluated whether age of the specimen and seasonality affected our measurements by including the number of years since collection and the time of year in separate analyses. We assessed seasonality by assigning each specimen to a warm or cool time of year based on the month of collection, where we considered April to October as “warm” and November to March as “cool.” Preliminary analyses revealed that habitat preferences were equally represented in the warm and cool seasons (*χ* ^2^ = 6.20, *P* = 0.10). Using the full dataset, we also assessed whether pelage thickness differed among species with different habitat preferences using an analysis of variance. Finally, we also assessed whether pelage thickness was associated with age of the specimen using an ANCOVA.

For the heat flux models, we used a paired *t*‐test to determine whether heating costs differed significantly between simulations with the observed and average conductivity between replicated sites. We selected the *t*‐test because the data were normally distributed for both diurnal (Shapiro‐Wilk; *W* = 0.96, *P* = 0.90) and nocturnal species (Shapiro‐Wilk; *W* = 0.99, *P* = 0.75).

## Results

**Figure 1:**
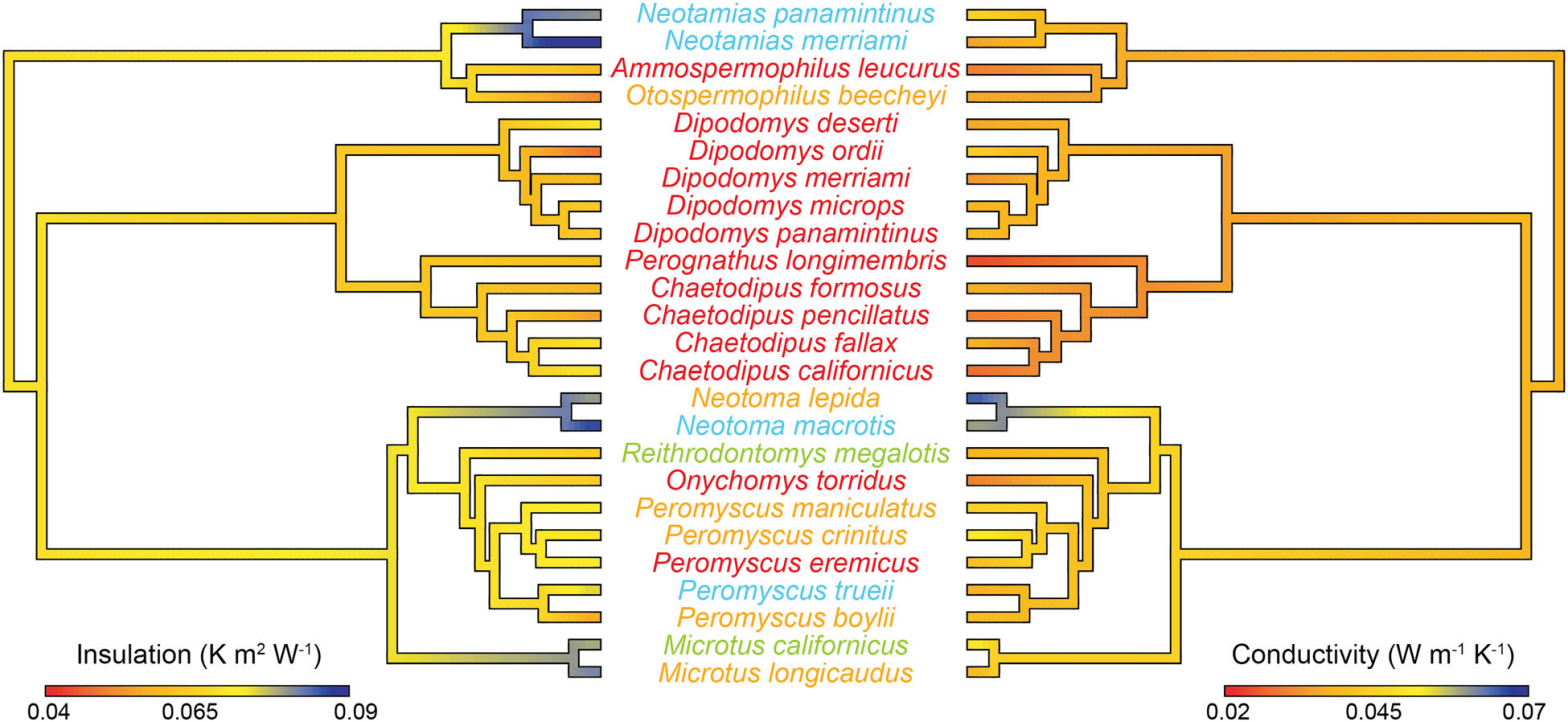
Evolutionary lability in the thermal properties of desert rodent pelage. Insulation and conductivity of desert rodent pelage are mapped onto reciprocally facing phylogenies to illustrate the associated evolution of the two traits. Colors depict continuous trait values, with high values for both traits illustrated in blue and low values in red. The color of the species’ names indicates habitat preferences (red = arid, green = grassland, yellow = generalist, blue = woodland).

Thermal properties of mammal pelage varied widely across desert rodents (Fig. 1). Insulation ranged between 0.044 K m^2^/W for *Dipodomys ordii* and 0.075 K m^2^/W for *Neotamias merriami*, whereas conductivity varied between 0.019 W/m/K for *Perognathus longimembris* to 0.071 W/m/K for *Neotoma lepida*. Phylogenetic analyses indicated that pelage conductivity was positively associated with pelage length (Table S3) and was much lower in arid than in grassland, woodland, and generalist species (Fig. S3). Pagel's *λ* indicated a lack of phylogenetic signal (*λ* = 0.0; *P*‐value for 0 = 1; *P*‐value for 1 = 0.01). Body mass and activity type were uninformative (Table S3). We did not find evidence for phylogenetic signal for conductivity of pelage (median *λ* = 0; median *P‐*value for 1 = 0.012; median *P*‐value for 0 = 1.0) when incorporating the phylogenetic uncertainty.

Insulation was positively related to pelage thickness (Fig. S4; Table S3), although the relationship was marginally significant, and insulation did not differ by habitat preference (Table S3). Species inhabiting woodlands exhibited the highest insulation (Fig. S4), although these differences were not statistically significant. Thus, arid specialists exhibited similar degrees of thermal insulation despite having substantially lower conductivity. Pelage length, body mass, and activity type were not associated with pelage insulation (Table S3). Pelage insulation also exhibited patterns consistent with evolutionary lability. We found no evidence for phylogenetic signal in insulation (*λ* = 0.0; *P*‐value for 0 = 1; *P*‐value for 1 < 0.001). We did not find evidence for phylogenetic signal for conductivity of pelage (median *λ* = 0; median *P‐*value for 1 < 0.001; median *P*‐value for 0 = 1.0) when incorporating the phylogenetic uncertainty. Together, the results from both conductivity and insulation are consistent with analyses using the consensus tree, indicating that the consensus tree approximated the observed variation in topology across the phylogenies. Due to the lack of phylogenetic signal, we focused on the full dataset consisting of all specimens.

**Figure 2:**
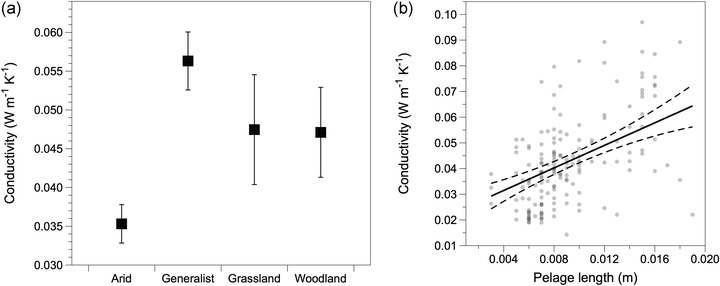
Effects of habitat preferences and pelage length on the thermal conductivity of mammal pelage. (a) Arid specialists exhibited the lowest thermal conductivity of the pelage among habitat preferences and (b) pelage length was positively correlated with conductivity.

Our results for conductivity and insulation were also qualitatively consistent with the phylogenetic and nonphylogenetic analyses. Similar to phylogenetic analyses, conductivity varied significantly by habitat preferences and pelage length (Fig. 2; Table S3). Arid specialists exhibited substantially lower conductivity than any other habitat preference (Fig. 2), and conductivity was positively associated with pelage length (Fig. 2). Insulation was significantly affected by pelage thickness and habitat preferences (Table S3). Although these variables were not statistically significant in the phylogenetic analysis, the differences among habitat preferences and association with pelage thickness were highly consistent between the phylogenetic and nonphylogenetic analyses (Fig. S4). The discrepancy between phylogenetic and nonphylogenetic analyses likely results from the low sample size in the former analysis. Pelage thickness also differed by habitat preferences (*P* < 0.001, *ω* ^2^ = 0.38), with arid species exhibiting the thinnest pelage among habitat groups (Fig. 4). Age of the museum specimen was not associated with conductivity (*P* = 0.20, *ω* ^2^ < 0.01) and was marginally associated with insulation (*P* = 0.06, *ω* ^2^ = 0.01). Season of collection did not influence conductivity (*P* = 0.73, *ω* ^2^ < 0.01) but did have an influence on insulation (*P* = 0.03, *ω* ^2^ = 0.02), with specimens collected during warmer months exhibiting less thermal insulation. Pelage thickness was also not associated with age of the specimen (*P* = 0.86, *ω* ^2^ < 0.01).

Heat flux simulations demonstrated that the observed variation in pelage conductivity significantly reduced thermoregulatory heating costs compared to the average conductivity (nocturnal: *t* = 74.70, *P* < 0.001; diurnal: *t* = 88.86, *P* < 0.001). Simulations with average pelage conductivity exhibited 14.5% higher heating costs compared to simulations with the observed conductivity (Fig. 5a). These differences were highly consistent for each month of the year, with the greatest absolute differences occurring in the cooler, winter months (Fig. 5b; range: 14.3%–15.0%). Diurnal species experienced a very similar increase in heating costs relative to the observed conductivity (Fig. S5; average = 14.6%). The differences were also consistent when incorporating daily torpor into the simulations (Fig. S6). Including torpor also reduced annual heating costs by 37.1% for nocturnal species and 46.2% for diurnal species relative to simulations without torpor. Heating costs for diurnal species were lower than nocturnal species (without torpor: 11.1%; with torpor: 24.0%). The reduction in heating costs were also consistent upon correcting pelage conductivity based on fresh specimens (16.2% for both diurnal and nocturnal species).

Finally, species exhibited substantial intraspecific variation in thermal conductivity, suggesting limited evidence for intrinsic constraints (Fig. 6).

## Discussion

**Figure 3:**
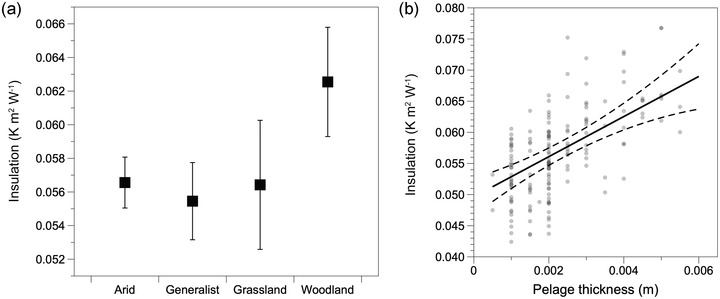
Effects of habitat preferences and pelage thickness on the thermal insulation of mammal pelage. (a) Woodland species exhibited the highest thermal insulation, and insulation was similar among the other habitat groups. (b) Pelage thickness was positively correlated with insulation.

**Figure 4:**
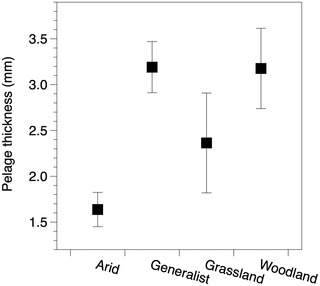
Arid species exhibited the lowest pelage thickness. Solid squares represent adjusted means, which are plotted with 95% confidence intervals.

We identified labile evolution in the thermal properties of pelage among a community of desert rodents and found support for selection favoring lower conductivity of pelage in arid specialists due to reduced metabolic expenditure. Rodents that prefer arid environments had substantially lower thermal conductivity relative to species with other habitat preferences (Fig. 2). Thermal conductivity among generalist, woodland, and grassland species was similar, suggesting that the pelage among these groups has similar thermal properties. Arid species exhibited similar insulation as generalists and grassland species (Fig. 3) despite having substantially thinner pelage (Fig. 4). The difference in thermal conductivity among habitat preferences and metabolic savings imparted by the low thermal conductivity suggest selective pressure from the environment drove adaptive evolution in the thermal properties of pelage in arid specialists. Moreover, the high degree of variability within species and overlap among species indicates intrinsic constraints were unlikely to be an important factor driving pelage evolution. Taken together, arid species have the thinnest pelage, which still provides the same insulation as generalists and grassland species due to the evolution of significantly lower conductivity. The combination of thinner pelage and effective insulation suggests that pelage plays an important role in adapting to hot and dry conditions while simultaneously maintaining insulation. These results indicate pelage conductivity as an important target of selection for reducing thermoregulatory heating costs.

**Figure 5:**
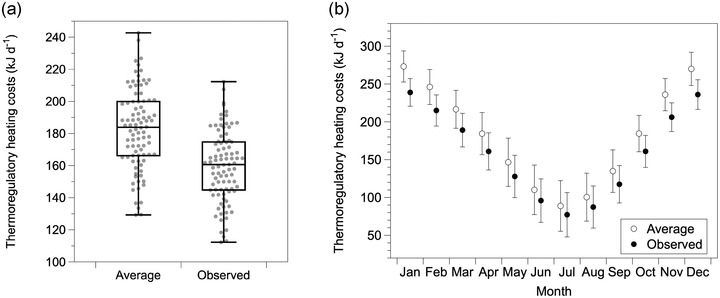
Heat flux simulations reveal potential for selection to favor lower thermal conductivity in arid specialists. (a) The difference in average daily heating costs between simulations using the observed variation in thermal conductivity of pelage compared with the average conductivity (i.e., average conductivity from generalists, grassland, and woodland species). Points represent heating costs for each site in the Mojave Desert assuming species are nocturnal. (b) Average monthly differences in heating costs for the observed conductivity (solid) compared with the hypothetical conductivity (open). Means are plotted with standard deviations.

**Figure 6:**
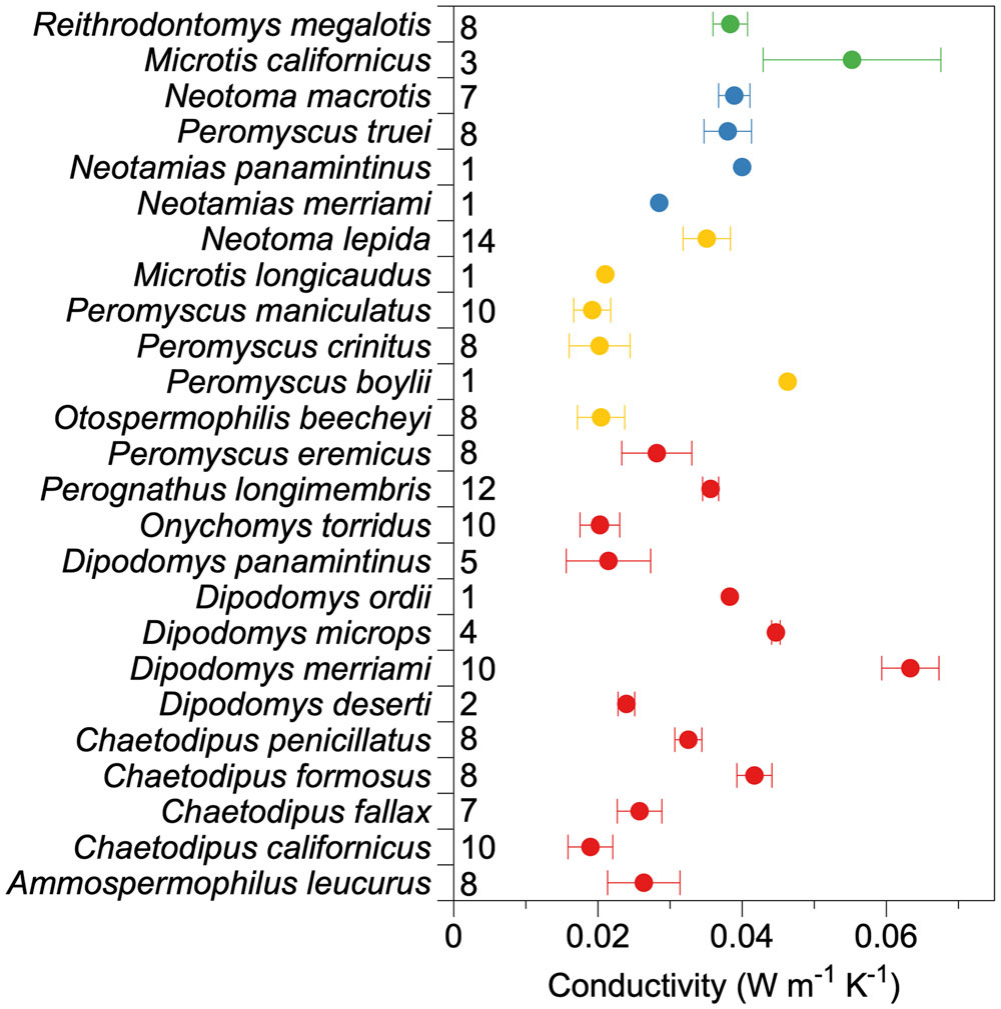
Intraspecific variation indicates lack of intrinsic constraints. Intraspecific variation in the thermal conductivity of pelage for the desert rodents with habitat preferences (red = arid, green = grassland, yellow = generalist, blue = woodland). Means with standard error are shown. Sample sizes for each species are shown to the right of each species abbreviation. The overlap between species suggests a lack of intrinsic constraints driving evolution of pelage conductivity.

### PELAGE BALANCES COOLING AND INSULATION

Pelage provides an insulative barrier between the skin and the environment, but this barrier may become disadvantageous in hot environments or when individuals are very active. Pelage acts to reduce heat flux by trapping air within the pelage, and thicker pelage provides more insulation by trapping more air (Walsberg 1991; Boyles and Bakken 2007). Desert mammals can experience cold temperatures during winter months of the year or in cool microhabitats (such as underground) that increase metabolic heat production to meet homeothermic requirements (Goodfriend et al. 1991; Riddell et al. 2021). Thus, maintaining an adequate layer of insulation is critical for keeping metabolic costs low, particularly when animals are active and not in a state of torpor. However, thick and insulative pelage impedes the movement of wind through pelage, lowering the rates of convective cooling (Tregear 1965; Boyles and Bakken 2007). Our results suggest that arid species have adapted to hot, arid conditions by producing thinner pelage with low conductivity, which promotes convective cooling while simultaneously maintaining the insulative potential of pelage. Arid species are thus provided the benefit of sufficient insulation when temperatures are cool and convective cooling when conditions are hot, such as during activity on the surface (Murray and Smith 2012; Levy et al. 2016). Our results provide the first example of how desert mammals have evolved to balance the trade‐off between maintaining insulation while simultaneously promoting the potential for convective cooling. The mechanisms by which conductivity evolves, however, are not well understood.

The most common way that mammals increase insulation is by growing or developing longer, denser pelage (Hammel 1955; Davis and Birkebak 1975; Webb and McClure 1988). Hair density in particular plays a fundamental role in determining the thermal insulation of pelage (Tregear 1965). The mechanisms underlying changes in the conductivity of hair are less clear. In arctic mammals, winter hairs have thick upper shafts consisting of air‐filled cells, whereas summer hairs are thinner (Russell and Tumlison 1996). In theory, air‐filled cells should reduce the conductivity of hair because air has much lower conductivity than pelage (Clement et al. 1981). However, air‐filled cells can also contribute to crypsis during the winter by reflecting more light (Russell and Tumlison 1996), although these hypotheses are not mutually exclusive. Conductivity might also be sensitive to the biochemical composition of hair, such as the type of keratin or lipids (Alibardi 2012). Changes in the properties of individual hairs might also be related to convective cooling. The thickness of hairs—even the thickness of upper or lower medullas—can have implications for convective cooling by influencing whether air flows around or through pelage (Russell and Tumlison 1996). Hair bends more easily when the lower medulla is thinner, promoting the flow of air around the pelage rather than through it. Conducting these types of mechanistic studies may reveal important insight into constraints on pelage evolution.

### EVOLUTIONARY POTENTIAL OF PELAGE

Our study indicates that the insulation and conductivity of small mammals is phylogenetically labile. We focused on rodents that inhabit the Mojave Desert, which consists roughly of 34 species (Riddell et al. 2021). Of these, 25 were represented as museum specimens for measurement. Despite the low sample size, phylogenetic analyses with more than 20 species can detect phylogenetic dependence in more than 90% of analyses (Freckleton et al. 2002), providing confidence in our conclusion of lability. Habitat preferences were also not evenly dispersed across our phylogeny, with arid species concentrated within *Chaetodipus* and *Dipodomys* (Fig. 1). However, arid species were also found outside of these groups, including *A. leucurus*, *Onychomys torridus*, and *Peromyscus eremicus*—all of which exhibited thin pelage with low conductivity and average insulation. The evolutionary lability of pelage can also be illustrated by the wide variety of hair types and diversity in biochemical properties (Clement et al. 1981; Alibardi 2012). Thus, our results suggest that the conductivity of the pelage is not constrained by relatedness, and variation in the thermal properties of pelage is driven by selective pressures associated with the environment. Given the broad temporal and spatial scale over which mammal specimens are collected, they make an excellent model system to understand pelage evolution.

Our results indicate that the thermal conductivity of the pelage is an important target of selection for arid specialists. There are many physiological, behavioral, and morphological traits that can influence the thermoregulatory costs for homeothermy. For instance, mammals can adjust heat loss by dynamically perfusing blood through the skin or acclimating to the thermal environment by adjusting the density of capillaries in the skin (Sealander 1951). Similarly, many desert species exhibit nocturnality and fossoriality—both considered important behavioral adaptations for surviving in deserts (Schmidt‐Nielsen 1964). The enlargement of certain appendages, such as ears, can improve heat dissipation and aid in survival (Knight and Skinner 1981). Thus, there are many aspects of desert rodent biology, including the density of the pelage and use of piloerection, that may still be important for understanding how selection reduces thermoregulatory costs or improves thermoregulatory efficiency. However, our study indicates that the thermal properties of the pelage are an important target of selection among the many traits related to thermoregulation.

### USE OF MAMMAL MUSEUM SPECIMENS FOR STUDY OF THERMAL PROPERTIES

Natural history museums offer a rich resource for studying ecology and evolution (Shaffer et al. 1998; Suarez and Tsutsui 2004; Tingley and Beissinger 2009; McLean et al. 2016). Mammal specimens are often well‐represented in museums and provide a window into responses to environmental change over time (Terry 2010; Terry et al. 2017). Our results indicate that flat skins of mammal pelage can be used to identify species‐level differences in the thermal properties of pelage. We assessed these differences by building an instrument that was capable of taking nondestructive measurements (Fig. S1). Similar studies often remove a sample of pelage from the skin to measure thermal conductance, destroying the specimen. We developed our novel instrument based on previous published experiments to measure comparable estimates of conductance while also preserving the collection for future use.

The drying process associated with the preparation of museum skins resulted in specimens with slightly higher thermal insulation than freshly prepared specimens, although this effect did not appear to influence our ability to distinguish relative differences in thermal properties of pelage among species. We also found that relationships between the thermal properties and pelage characteristics were consistent with the literature and comparable to other studies (Fig. S2). Moreover, the age of the specimen (which ranged from 2 to 98 years old) did not have a large effect on conductivity or insulation. Specimen age had a marginal effect on insulation, but this effect was confounded by site of collection. Thus, we were unable to determine whether this small effect reflected the age of the specimen or geographic variation in pelage properties. Given that age of the specimen had no effect on conductivity of the pelage, we believe museum specimens of mammal skins provide a valuable resource for understanding the ecology and evolution of thermal adaptation across space and time, which can contribute to understanding the impact of climate change on small mammal communities.

### THE ROLE OF PELAGE DURING CLIMATE CHANGE

The ecological effects of climate change on small mammal communities are likely to be variable and difficult to predict. In response to climate change, some species have shifted their elevational or latitudinal ranges to track cooler environments (Moritz et al. 2008; Beever et al. 2011), whereas others have shifted in unexpected ways or not at all (Moritz et al. 2008; Rowe et al. 2010; Rowe et al. 2015; Wen et al. 2017). The small mammal community associated with this study has remained stable in the Mojave Desert over the last century of climate change, likely due to the ability of species to seek cool subterranean microhabitats (Riddell et al. 2021). However, small mammals are expected to become more exposed to hot, dry conditions from climate change (Lovegrove et al. 2014), opening the potential for selection to act on phenotypes that influence heat flux.

The properties of pelage exhibit a high degree of seasonal flexibility, including changes in density, length, diameter of hair, and presence of air‐filled cells (Hart and Heroux 1953; Hart 1956; Walsberg 1991; Jofré and Caviedes‐Vidal 2003; Boyles and Bakken 2007). Similar to cold‐adapted species, our study revealed seasonal flexibility in pelage insulation. Changes in pelage properties are not limited to insulation or conductivity. Pelage thickness determines how easily solar radiation penetrates the pelage (Walsberg and Schmidt 1989; Walsberg and Wolf 1995; Walsberg et al. 1997), and dark pelage in particular can act as a “heat shield” by trapping solar radiation at the surface of the pelage (Walsberg and Wolf 1995). The high degree of flexibility in these characteristics suggests that pelage could play an important role in adapting to climate change. Similarly, these complex characteristics of pelage can also improve models that use biophysics to forecast the physiological impact of climate change (Riddell et al. 2021). Thus, our study underscores the need to focus on the potential for the properties of pelage to benefit small mammals in the face of greater physiological challenges imposed by climate change.

## Conclusions

We demonstrated the potential for adaptive evolution of pelage and identified specific properties of pelage as an important target of selection for minimizing thermoregulatory costs in desert rodents. Our study reveals the importance of the local environment in driving the evolution of pelage properties, such as conductivity. As a consequence, we identified the potential for adaptation of pelage to mitigate the increasingly important need to stay cool during climate change while also maintaining a sufficient buffer from heat loss. We also demonstrated that museum specimens of mammal skins can be used to understand the thermal properties of pelage, providing insight into the ecology and evolution of small mammals. Museum collections offer an important opportunity to identify how mammals respond to variation in environmental conditions across space and time by modifying the thermal properties of pelage.

## AUTHOR CONTRIBUTIONS

ER measured the thermal properties of fur, conducted the analyses, and developed the heat flux simulations. ER, JP, and SB conceived the project and wrote the manuscript.

## CONFLICT OF INTEREST

The authors declare no conflict of interest.

## DATA ARCHIVING

All data and code have been made freely available on Data Dryad (https://doi.org/10.5061/dryad.0zpc8671g).

Associate Editor: T. Kohlsdorf

Handling Editor: T. Chapman

## Supporting information


**Table S1**. List of specimen catalog numbers used to estimate the thermal conductance of the pelage.
**Table S2**. Morphological traits used in the heat flux model to estimate the thermoregulatory costs associated with different values of conductivity. The table contains the trait, values, and units. The data for these values were discussed at length in Riddell et al. (2021).
**Table S3**. Non‐phylogenetic approach exhibits similar responses to phylogenetic approaches for insulation and conductivity. In the phylogenetic analysis for conductivity, habitat preferences (arid, grassland, woodland, generalist) and pelage length affected the conductivity of mammal pelage. Body mass and activity type (diurnal or nocturnal) did not affect conductivity. In the phylogenetic analysis for insulation, we found a trend between insulation and pelage thickness. We did not detect differences based on habitat preferences, body mass, activity type, and pelage length. Non‐phylogenetic analyses indicate that conductivity was significantly associated with habitat preferences and pelage length, and insulation was significantly associated with habitat preferences and pelage thickness.
**Fig. S1**. Schematic of heat flux device used to measure thermal insulation. The various components of the heat flux device are labelled. Not shown are the water pump, water bath, incubator, and additional thermocouples. The distance between the heat flux transducer, specimen, and platform are exaggerated; these components were pressed tightly together during measurements.
**Fig. S2**. Consistency in pelage thickness and insulation relationship from museum specimens. Pelage insulation of museum specimens exhibited variation consistent with empirically‐based expectations from a classical study on the thermal properties of mammal pelage by Scholander et al. (1950). Solid black points (with standard error) are from the 25 species from this study and open points from Scholander et al. (1950).
**Fig. S3**. Partial effects on the thermal conductivity of mammal pelage for (A) pelage length and (B) habitat preferences with standard errors based on residuals of PGLS analyses and model estimates, respectively.
**Fig. S4**. Partial effect of thickness on the thermal insulation of pelage for (A) pelage thickness and (B) habitat preferences (means with standard errors) from residuals of corresponding PGLS analyses and model estimates, respectively.
**Fig. S5**. Heat flux simulations for diurnal species reveal potential for selection to favor lower thermal conductivity in arid specialists. (A) The difference in average daily heating costs between simulations using the observed variation in thermal conductivity of pelage compared with the average conductivity (i.e., average conductivity from generalists, grassland, and woodland species). Points represent heating costs for each site in the Mojave Desert. (B) Average monthly differences in heating costs for the observed conductivity (solid) compared with the hypothetical conductivity (open). Means are plotted with standard deviations.
**Fig. S6**. Estimates of thermoregulatory heating costs that include torpor. The change in heating costs was consistent with models that did not include torpor for both (A) nocturnal and (B) diurnal species. Average monthly differences in heating costs for the observed conductivity (solid) compared with the hypothetical conductivity (open). Means are plotted with standard deviations.
