# Supplementary material for: Thermal adaptation of pelage in desert rodents balances cooling and insulation
Source: Evolution. 2022 Oct 18;76(12):3001–13. doi: 10.1111/evo.14643 (PMC10091991; doi:10.1111/evo.14643)
Supplement: Supplementary file 1 — Table S1. List of specimen catalog numbers used to estimate the thermal conductance of the pelage. Table S2. Morphological traits used in the heat flux model to estimate the thermoregulatory costs associated with different values of conductivity. The table contains the trait, values, and units. The data for these values were discussed at length in Riddell et al. (2021). Table S3. Non‐phylogenetic approach exhibits similar responses to phylogenetic approaches for insulation and conductivity. In the phylogenetic analysis for conductivity, habitat preferences (arid, grassland, woodland, generalist) and pelage length affected the conductivity of mammal pelage. Body mass and activity type (diurnal or nocturnal) did not affect conductivity. In the phylogenetic analysis for insulation, we found a trend between insulation and pelage thickness. We did not detect differences based on habitat preferences, body mass, activity type, and pelage length. Non‐phylogenetic analyses indicate that conductivity was significantly associated with habitat preferences and pelage length, and insulation was significantly associated with habitat preferences and pelage thickness. Fig. S1. Schematic of heat flux device used to measure thermal insulation. The various components of the heat flux device are labelled. Not shown are the water pump, water bath, incubator, and additional thermocouples. The distance between the heat flux transducer, specimen, and platform are exaggerated; these components were pressed tightly together during measurements. Fig. S2. Consistency in pelage thickness and insulation relationship from museum specimens. Pelage insulation of museum specimens exhibited variation consistent with empirically‐based expectations from a classical study on the thermal properties of mammal pelage by Scholander et al. (1950). Solid black points (with standard error) are from the 25 species from this study and open points from Scholander et al. (1950). Fig. S3. Partial effects on the ther [file EVO-76-3001-s001.docx]

**Table S1**. **List of specimen catalog numbers used to estimate the thermal conductance of the pelage**.

| Species | MVZ Catalog Number | Mass (g) | Date captured |
| --- | --- | --- | --- |
| *Ammospermophilus leucurus* | 28406 | 76 | 9/21/2018 |
| *Ammospermophilus leucurus* | 28428 | 108 | 9/21/2018 |
| *Ammospermophilus leucurus* | 28429 | 95 | 9/21/2018 |
| *Ammospermophilus leucurus* | 28430 | 74 | 9/21/2018 |
| *Ammospermophilus leucurus* | 28432 | 71 | 9/22/2018 |
| *Ammospermophilus leucurus* | 28544 | 93 | 10/8/2018 |
| *Ammospermophilus leucurus* | 28600 | 83 | 10/18/2018 |
| *Ammospermophilus leucurus* | 28601 | 99 | 10/18/2018 |
| *Chaetodipus californicus* | 88362 | 24 | 12/17/1938 |
| *Chaetodipus californicus* | 88363 | 25 | 12/18/1938 |
| *Chaetodipus californicus* | 88364 | 26 | 12/18/1938 |
| *Chaetodipus californicus* | 88365 | 22 | 12/18/1938 |
| *Chaetodipus californicus* | 88366 | 28 | 12/18/1938 |
| *Chaetodipus californicus* | 88367 | 27 | 12/18/1938 |
| *Chaetodipus californicus* | 88368 | 22.5 | 12/18/1938 |
| *Chaetodipus californicus* | 88369 | 24 | 12/18/1938 |
| *Chaetodipus californicus* | 89685 | 18.5 | 12/28/1939 |
| *Chaetodipus fallax* | 89690 | 15 | 12/31/1939 |
| *Chaetodipus fallax* | 90104 | 19 | 1/16/1940 |
| *Chaetodipus fallax* | 90105 | 18 | 1/16/1940 |
| *Chaetodipus fallax* | 90106 | 18 | 1/16/1940 |
| *Chaetodipus fallax* | 90107 | 15 | 1/16/1940 |
| *Chaetodipus fallax* | 90657 | 24 | 3/23/1940 |
| *Chaetodipus penicillatus* | 90109 | 20 | 1/15/1940 |
| *Chaetodipus penicillatus* | 90110 | 23 | 1/16/1940 |
| *Chaetodipus penicillatus* | 90111 | 19 | 1/16/1940 |
| *Chaetodipus penicillatus* | 90113 | 18 | 1/8/1940 |
| *Chaetodipus penicillatus* | 90656 | 17 | 3/23/1940 |
| *Chaetodipus californicus* | 85071 | 26 | 4/6/1939 |
| *Chaetodipus fallax* | 84370 | 17.5 | 7/31/1938 |
| *Chaetodipus formosus* | 28446 | 14 | 9/23/2018 |
| *Chaetodipus formosus* | 28447 | 18 | 9/23/2018 |
| *Chaetodipus formosus* | 28448 | 12 | 9/23/2018 |
| *Chaetodipus formosus* | 28624 | 19 | 10/22/2018 |
| *Chaetodipus formosus* | 28625 | 17 | 10/22/2018 |
| *Chaetodipus formosus* | 28626 | 18 | 10/22/2018 |
| *Chaetodipus formosus* | 28627 | 15 | 10/22/2018 |
| *Chaetodipus formosus* | 84372 | 16.5 | 8/7/1938 |
| *Chaetodipus penicillatus* | 85070 | 18 | 4/3/1940 |
| *Chaetodipus penicillatus* | 90714 | 19 | 4/4/1940 |
| *Chaetodipus penicillatus* | 99956 | 19 | 4/20/1941 |
| *Dipodomys deserti* | 27426 | 115 | 3/11/2017 |
| *Dipodomys deserti* | 27427 | 98 | 3/11/2017 |
| *Dipodomys merriami* | 28408 | 32 | 9/21/2018 |
| *Dipodomys merriami* | 28410 | 41 | 9/21/2018 |
| *Dipodomys merriami* | 28433 | 31 | 9/22/2018 |
| *Dipodomys merriami* | 28434 | 32 | 9/22/2018 |
| *Dipodomys merriami* | 28435 | 32 | 9/22/2018 |
| *Dipodomys merriami* | 28436 | 31 | 9/22/2018 |
| *Dipodomys merriami* | 28628 | 33 | 10/22/2018 |
| *Dipodomys merriami* | 28629 | 31 | 10/22/2018 |
| *Dipodomys merriami* | 28630 | 33 | 10/22/2018 |
| *Dipodomys merriami* | 28631 | 34 | 10/22/2018 |
| *Dipodomys microps* | 28453 | 43 | 9/23/2018 |
| *Dipodomys microps* | 28454 | 43 | 9/23/2018 |
| *Dipodomys microps* | 28455 | 47 | 9/23/2018 |
| *Dipodomys microps* | 28456 | 48 | 9/23/2018 |
| *Dipodomys ordii* | 28407 | 40 | 9/21/2018 |
| *Dipodomys panamintinus* | 28409 | 77 | 9/21/2018 |
| *Dipodomys panamintinus* | 28411 | 55 | 9/21/2018 |
| *Dipodomys panamintinus* | 28412 | 78 | 9/21/2018 |
| *Dipodomys panamintinus* | 28431 | 73 | 9/21/2018 |
| *Dipodomys panamintinus* | 28437 | 67 | 9/21/2018 |
| *Microtis californicus* | 89903 | 58 | 1/4/1940 |
| *Microtis californicus* | 89904 | 60 | 1/4/1940 |
| *Microtis californicus* | 90271 | 97 | 1/11/1940 |
| *Microtis longicaudus* | 88683 | NA | 7/13/1939 |
| *Neotoma lepida* | 27425 | 128 | 3/11/2018 |
| *Neotoma lepida* | 27434 | 71 | 3/12/2017 |
| *Neotoma lepida* | 27435 | 81 | 3/12/2017 |
| *Neotoma lepida* | 27436 | 110 | 3/12/2017 |
| *Neotoma macrotis* | 88552 | 195 | 12/17/1938 |
| *Neotoma macrotis* | 88553 | 230 | 12/17/1938 |
| *Neotoma macrotis* | 88554 | 220 | 12/17/1938 |
| *Neotoma macrotis* | 88555 | 230 | 12/17/1938 |
| *Neotoma macrotis* | 88556 | 250 | 12/17/1938 |
| *Neotoma macrotis* | 88557 | 195 | 12/18/1938 |
| *Neotoma lepida* | 28413 | 108 | 9/21/2018 |
| *Neotoma lepida* | 28414 | 131 | 9/21/2018 |
| *Neotoma lepida* | 28415 | 152 | 9/21/2018 |
| *Neotoma lepida* | 28416 | 112 | 9/21/2018 |
| *Neotoma lepida* | 28417 | 115 | 9/21/2018 |
| *Neotoma lepida* | 28541 | 92 | 10/7/2018 |
| *Neotoma lepida* | 28542 | 110 | 10/7/2018 |
| *Neotoma lepida* | 28543 | 112 | 10/7/2018 |
| *Neotoma lepida* | 28587 | 102 | 10/18/2018 |
| *Neotoma lepida* | 28588 | 93 | 10/18/2018 |
| *Neotoma macrotis* | 226032 | 170 | 5/16/2005 |
| *Onychomys torridus* | 88418 | 22 | 12/31/1938 |
| *Onychomys torridus* | 88423 | 17 | 12/30/1938 |
| *Onychomys torridus* | 88424 | 20 | 12/31/1938 |
| *Onychomys torridus* | 90171 | 24 | 1/14/1940 |
| *Onychomys torridus* | 90172 | 22 | 1/14/1940 |
| *Onychomys torridus* | 90173 | 22 | 1/14/1940 |
| *Onychomys torridus* | 90174 | 25 | 1/12/1940 |
| *Onychomys torridus* | 28426 | 13 | 9/21/2018 |
| *Onychomys torridus* | 85099 | 24 | 4/3/1939 |
| *Onychomys torridus* | 85100 | 29 | 4/3/1939 |
| *Otospermophilis beecheyi* | 89625 | NA | 1/4/1940 |
| *Otospermophilis beecheyi* | 90649 | 530 | 3/21/1940 |
| *Otospermophilis beecheyi* | 90650 | 475 | 3/21/1940 |
| *Otospermophilis beecheyi* | 99904 | 450 | 3/8/1941 |
| *Otospermophilis beecheyi* | 99905 | 400 | 3/8/1941 |
| *Otospermophilis beecheyi* | 99906 | 660 | 1/4/1940 |
| *Otospermophilis beecheyi* | 99907 | 470 | 1/4/1940 |
| *Otospermophilis beecheyi* | 99908 | 370 | 6/22/1941 |
| *Perognathus longimembris* | 84361 | 7 | 8/14/1938 |
| *Perognathus longimembris* | 84362 | 6.5 | 8/14/1938 |
| *Perognathus longimembris* | 85050 | 9 | 4/3/1939 |
| *Perognathus longimembris* | 85051 | 8 | 4/3/1939 |
| *Perognathus longimembris* | 85052 | 8 | 4/3/1939 |
| *Perognathus longimembris* | 85053 | 8 | 4/3/1939 |
| *Perognathus longimembris* | 85054 | 8 | 4/3/1939 |
| *Perognathus longimembris* | 85060 | 10 | 4/3/1939 |
| *Perognathus longimembris* | 85061 | 9 | 4/3/1939 |
| *Perognathus longimembris* | 85062 | 9 | 4/3/1939 |
| *Perognathus longimembris* | 85063 | 10 | 4/3/1939 |
| *Perognathus longimembris* | 85064 | 10 | 4/3/1939 |
| *Peromyscus boylii* | * | 24 | 11/16/1975 |
| *Peromyscus eremicus* | 27428 | 21 | 3/11/2017 |
| *Peromyscus eremicus* | 209661 | NA | 1/5/2022 |
| *Peromyscus eremicus* | 209663 | NA | 1/5/2022 |
| *Peromyscus eremicus* | 209669 | NA | 1/5/2022 |
| *Peromyscus eremicus* | 209670 | NA | 1/5/2022 |
| *Peromyscus eremicus* | 209673 | NA | 1/5/2022 |
| *Peromyscus truei* | 215457 | NA | 1/5/2022 |
| *Peromyscus crinitus* | 28423 | 15 | 9/21/2018 |
| *Peromyscus crinitus* | 28424 | 13 | 9/21/2018 |
| *Peromyscus crinitus* | 28425 | 13 | 9/21/2018 |
| *Peromyscus crinitus* | 28438 | 13 | 9/21/2018 |
| *Peromyscus crinitus* | 28449 | 13 | 9/23/2018 |
| *Peromyscus crinitus* | 28450 | 14 | 9/23/2018 |
| *Peromyscus crinitus* | 28452 | 13 | 9/23/2018 |
| *Peromyscus crinitus* | 28541 | 13 | 9/23/2018 |
| *Peromyscus eremicus* | 28573 | 18 | 10/14/2018 |
| *Peromyscus eremicus* | 28574 | 19 | 10/14/2018 |
| *Peromyscus maniculatus* | 28418 | 18 | 9/21/2018 |
| *Peromyscus maniculatus* | 28419 | 18 | 9/21/2018 |
| *Peromyscus maniculatus* | 28420 | 12 | 9/21/2018 |
| *Peromyscus maniculatus* | 28421 | 17 | 9/21/2018 |
| *Peromyscus maniculatus* | 28422 | 23 | 9/21/2018 |
| *Peromyscus maniculatus* | 28567 | 18 | 10/14/2018 |
| *Peromyscus maniculatus* | 28568 | 19 | 10/14/2018 |
| *Peromyscus maniculatus* | 28596 | 23 | 10/18/2018 |
| *Peromyscus maniculatus* | 28597 | 20 | 10/18/2018 |
| *Peromyscus maniculatus* | 28598 | 18 | 10/18/2018 |
| *Peromyscus truei* | 28563 | 14 | 10/13/2018 |
| *Peromyscus truei* | 28564 | 24 | 10/13/2018 |
| *Peromyscus truei* | 28565 | 26 | 10/13/2018 |
| *Peromyscus truei* | 28566 | 28 | 10/13/2018 |
| *Peromyscus truei* | 215447 | NA | 9/19/2022 |
| *Peromyscus truei* | 215448 | NA | 9/19/2022 |
| *Peromyscus truei* | 215456 | NA | 9/19/2022 |
| *Reithrodontomys megalotis* | 88425 | 8 | 12/17/1938 |
| *Reithrodontomys megalotis* | 88426 | 10.5 | 12/17/1938 |
| *Reithrodontomys megalotis* | 88427 | 9 | 12/17/1938 |
| *Reithrodontomys megalotis* | 88434 | 9 | 12/27/1938 |
| *Reithrodontomys megalotis* | 28427 | 10 | 9/21/2018 |
| *Reithrodontomys megalotis* | 28439 | 14 | 9/22/2018 |
| *Reithrodontomys megalotis* | 28440 | 12 | 9/22/2018 |
| *Reithrodontomys megalotis* | 28441 | 15 | 9/22/2018 |
| *Neotamias merriami* | 33433 | NA | 12/31/2023 |
| *Neotamias panamintinus* | 79950 | 51 | 1/7/1937 |

* catalog number not available, specimen came from the mammal exchange collection

NA = not available

**Table S2. Morphological traits used in the heat flux model to estimate the thermoregulatory costs associated with different values of conductivity.** The table contains the trait, values, and units. The data for these values were discussed at length in Riddell et al. (2021).

| **Trait** | **Value** | **Units** |
| --- | --- | --- |
| Mass | 45.392 | g |
| Conductivity | 0.0353 – 0.0503* | W m^-1^ K^-1^ |
| Dorsal pelage length | 0.0097 | m |
| Ventral pelage length | 0.0067 | m |
| Dorsal pelage depth | 0.0017 | m |
| Dorsal pelage length | 0.0017 | m |
| Body length | 0.1129 | m |
| Body width | 0.0339 | m |
| Body height | 0.0339 | m |
| Fiber density | 11,200 | fibers cm^-2^ |
| Emissivity | 0.97 | dimensionless |

* depended on scenario (average or observed)

**Table S3.** **Non-phylogenetic approach exhibits similar responses to phylogenetic approaches for insulation and conductivity.** In the phylogenetic analysis for conductivity, habitat preferences (arid, grassland, woodland, generalist) and pelage length affected the conductivity of mammal pelage. Body mass and activity type (diurnal or nocturnal) did not affect conductivity. In the phylogenetic analysis for insulation, we found a trend between insulation and pelage thickness. We did not detect differences based on habitat preferences, body mass, activity type, and pelage length. Non-phylogenetic analyses indicate that conductivity was significantly associated with habitat preferences and pelage length, and insulation was significantly associated with habitat preferences and pelage thickness.

| **Phylogenetic analysis** | | | | | | | | |
| --- | --- | --- | --- | --- | --- | --- | --- | --- |
| ***Insulation*** |  |  |  | |  | |  | |
| *Variable* | *df* | F | Z | | *p* | |  | |
| Log_10_(Mass) | 1 | 0.67 | | 0.25 | | 0.42 | |  |
| Pelage length | 1 | 0.76 | | 0.30 | | 0.40 | |  |
| Activity type | 1 | 0.08 | | -0.75 | | 0.77 | |  |
| Habitat preference | 3 | 1.23 | | 0.42 | | 0.32 | |  |
| Pelage depth | 1 | 3.14 | | 1.33 | | 0.09 | |  |
| Residuals | 17 |  | |  | |  | |  |
| ***Conductivity*** |  |  |  | |  | |  | |
| Variable | *df* | F | Z | | *p* | |  | |
| Log_10_(Mass) | 1 | 0.45 | -0.01 | | 0.50 | |  | |
| Pelage length | 1 | 5.61 | 1.85 | | 0.03 | |  | |
| Activity type | 1 | 0.28 | -0.25 | | 0.61 | |  | |
| Habitat preference | 3 | 4.99 | 2.38 | | 0.01 | |  | |
| Residuals | 18 |  |  | |  | |  | |
| **Non-phylogenetic analysis** | | | | | | |  |  |
| ***Insulation*** |  |  |  | |  | |  |  |
| *Variable* | *df* | *F* | *p* | | *ω*^2^ | |  |  |
| Habitat preference | 3 | 5.26 | | 0.002 | | 0.05 | |  |
| Activity | 1 | 0.69 | | 0.408 | | 0.00 | |  |
| Pelage thickness | 1 | 22.5 | | <0.001 | | 0.17 | |  |
| log(Mass) | 1 | 0.15 | | 0.701 | | 0.02 | |  |
| Pelage length | 1 | 0.55 | | 0.460 | | 0.12 | |  |
| Residuals | 155 |  | |  | |  | |  |
| ***Conductivity*** |  |  |  | |  | |  |  |
| *Variable* | *df* | *F* | *p* | | *ω*^2^ | |  |  |
| Habitat preference | 3 | 28.1 | <0.001 | | 0.23 | |  |  |
| Activity | 1 | 0.13 | 0.813 | | 0.00 | |  |  |
| log(Mass) | 1 | 0.06 | 0.713 | | 0.16 | |  |  |
| Pelage length | 1 | 30.7 | <0.001 | | 0.17 | |  |  |
| Residuals | 156 |  |  | |  | |  |  |

**Fig. S1. Schematic of heat flux device used to measure thermal insulation.** The various components of the heat flux device are labelled. Not shown are the water pump, water bath, incubator, and additional thermocouples. The distance between the heat flux transducer, specimen, and platform are exaggerated; these components were pressed tightly together during measurements.


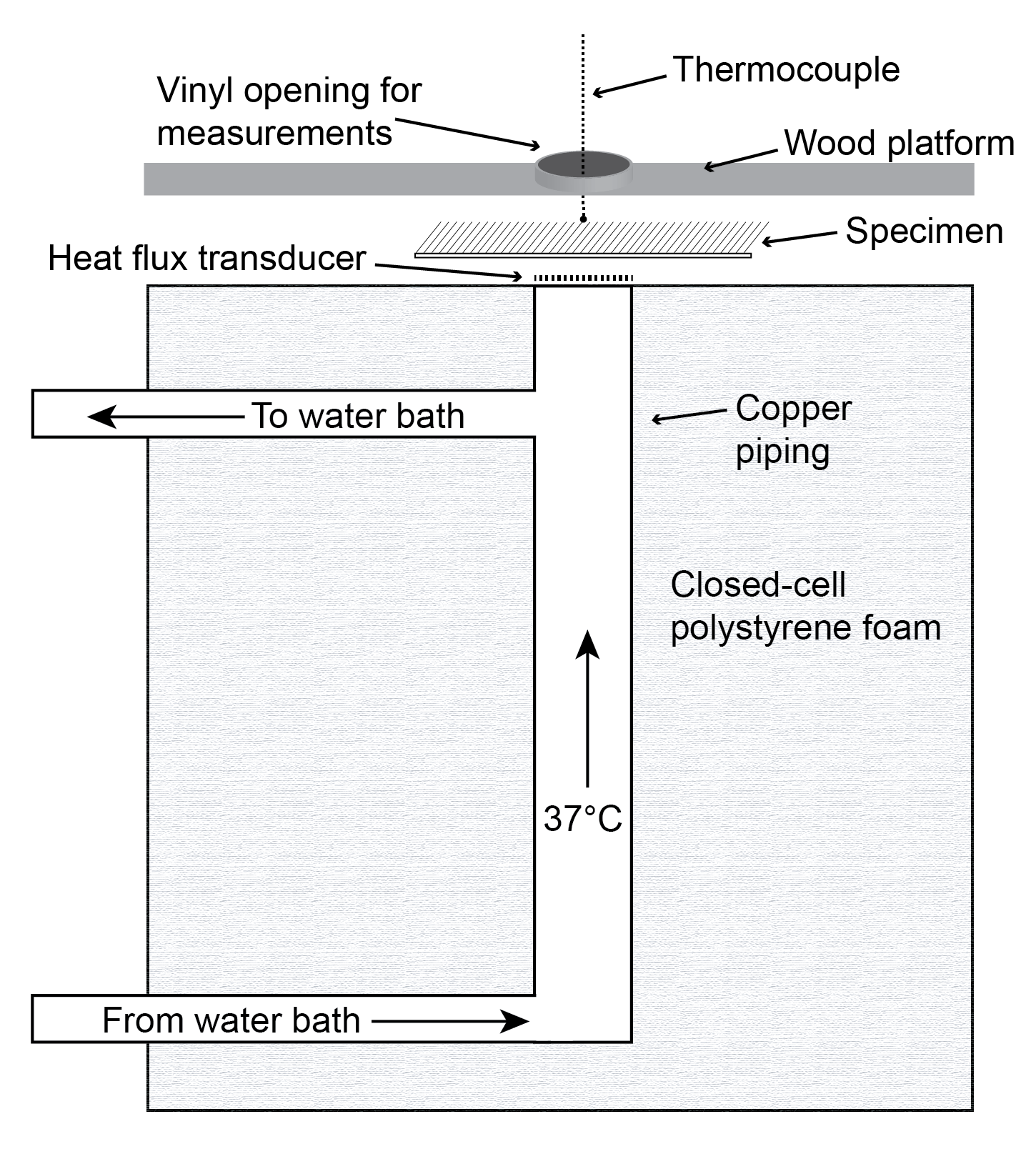


**Fig. S2.** **Consistency in pelage thickness and insulation relationship from museum specimens.** Pelage insulation of museum specimens exhibited variation consistent with empirically-based expectations from a classical study on the thermal properties of mammal pelage by Scholander *et al*. (1950). Solid black points (with standard error) are from the 25 species from this study and open points from Scholander *et al*. (1950).


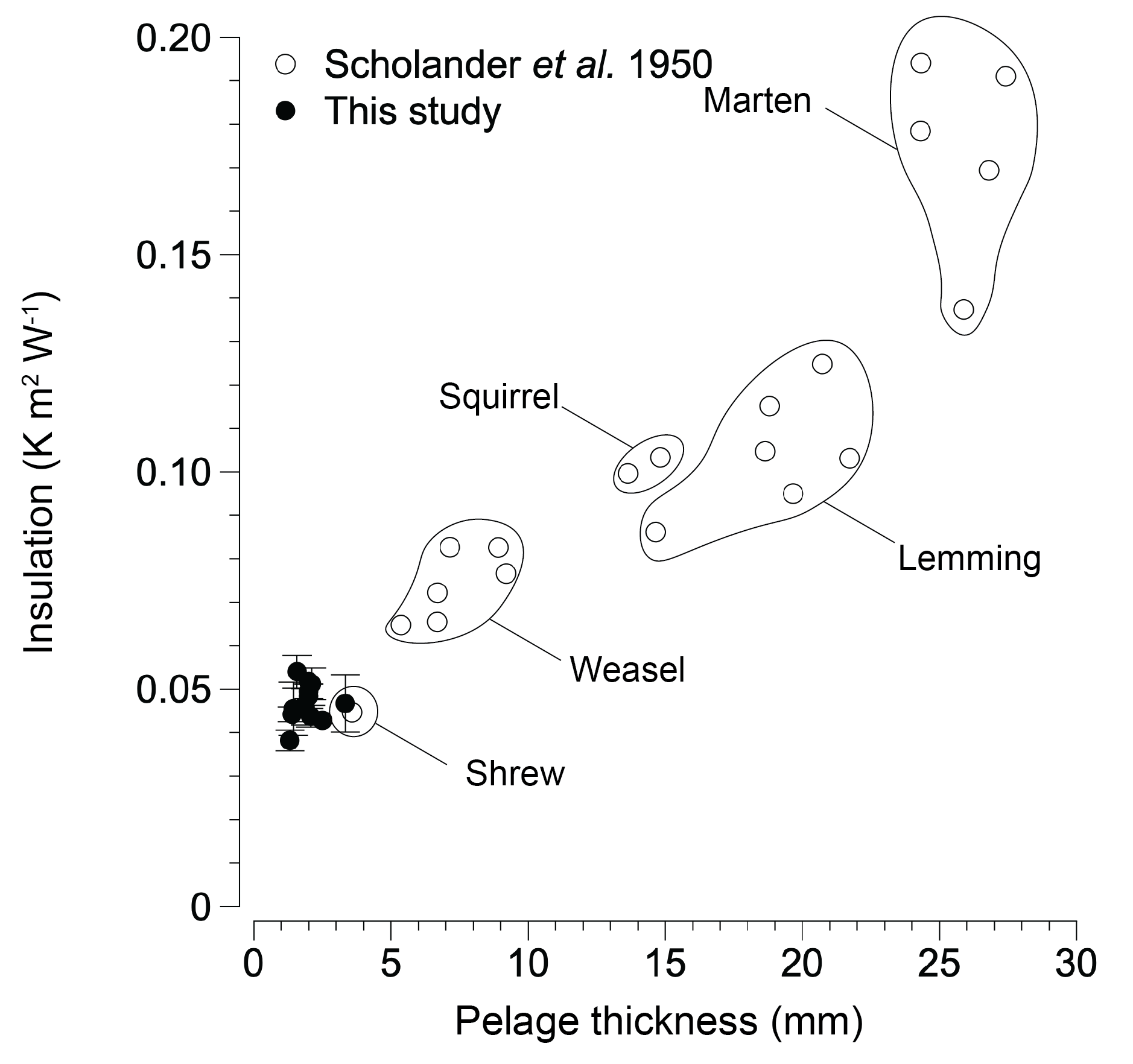


Fig. S3. **Partial effects on the thermal conductivity of mammal pelage** for (A) pelage length and (B) habitat preferences with standard errors based on residuals of PGLS analyses and model estimates, respectively.


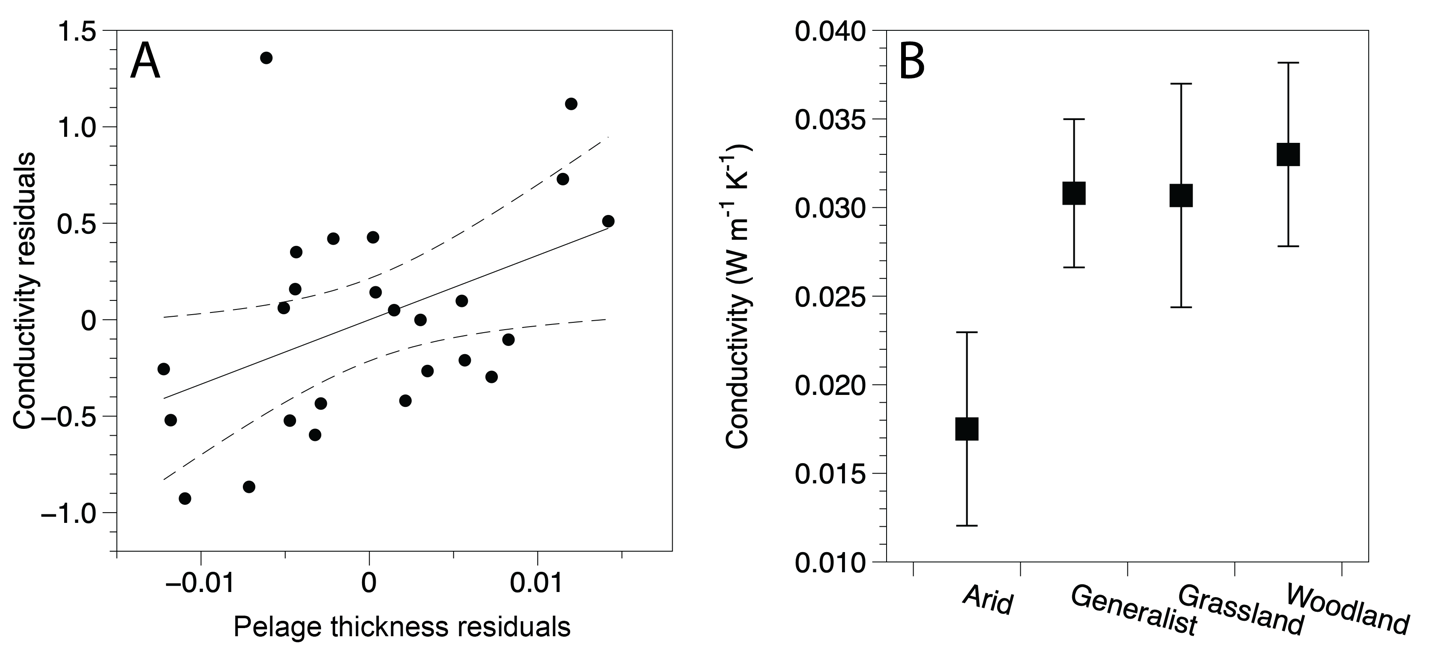


Fig. S4. **Partial effect of thickness on the thermal insulation of pelage** for (A) pelage thickness and (B) habitat preferences (means with standard errors) from residuals of corresponding PGLS analyses and model estimates, respectively.


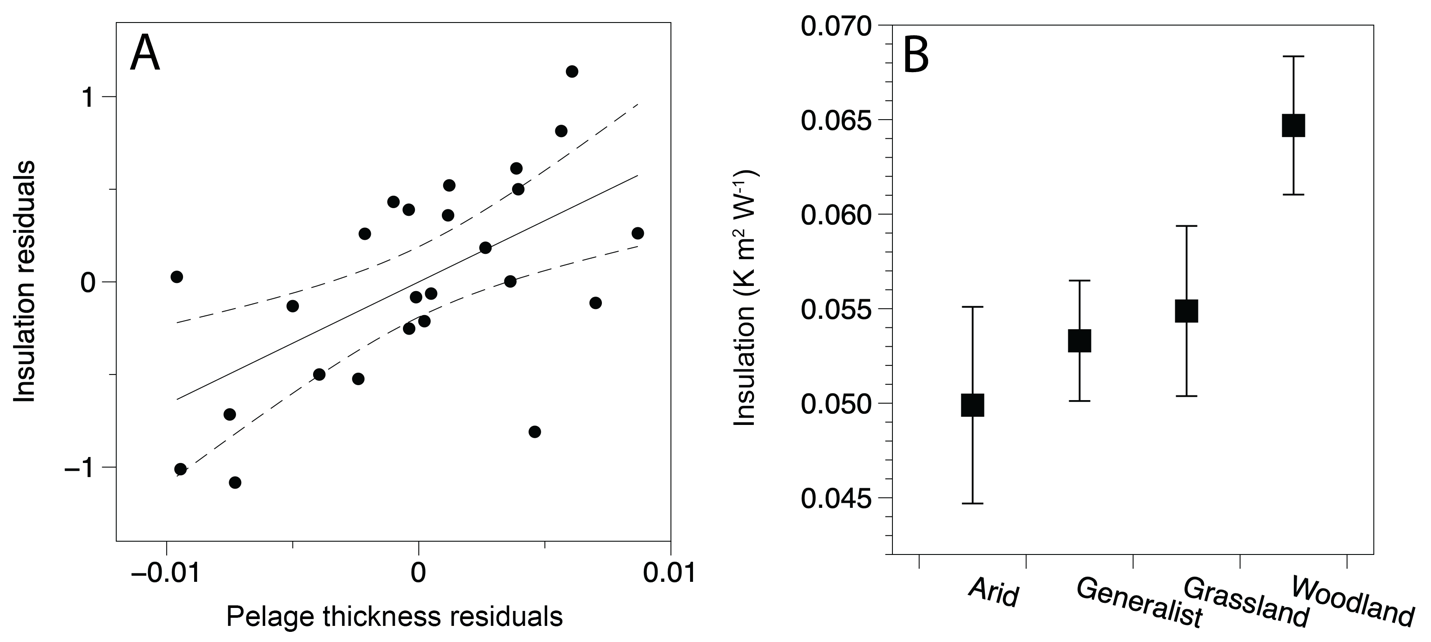


**Fig. S5.** **Heat flux simulations for diurnal species reveal potential for selection to favor lower thermal conductivity in arid specialists**. (A) The difference in average daily heating costs between simulations using the observed variation in thermal conductivity of pelage compared with the average conductivity (i.e., average conductivity from generalists, grassland, and woodland species). Points represent heating costs for each site in the Mojave Desert. (B) Average monthly differences in heating costs for the observed conductivity (solid) compared with the hypothetical conductivity (open). Means are plotted with standard deviations.

**
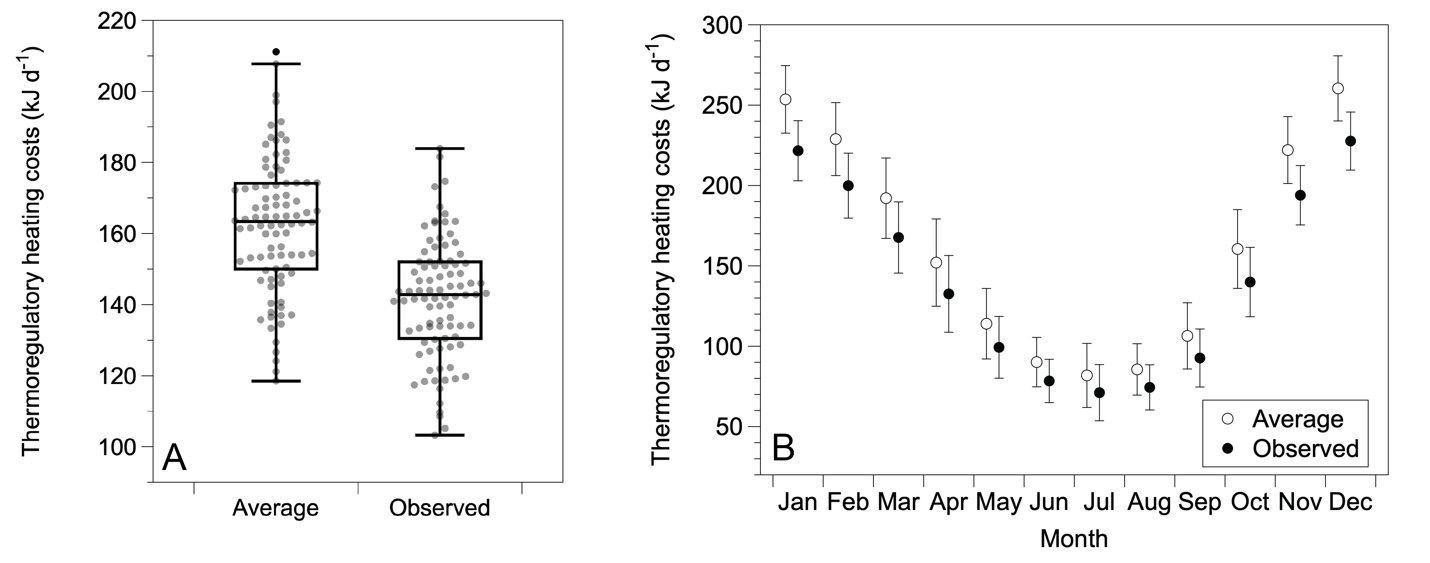
**

**Fig. S6.** **Estimates of thermoregulatory heating costs that include torpor**. The change in heating costs was consistent with models that did not include torpor for both (A) nocturnal and (B) diurnal species. Average monthly differences in heating costs for the observed conductivity (solid) compared with the hypothetical conductivity (open). Means are plotted with standard deviations.

**
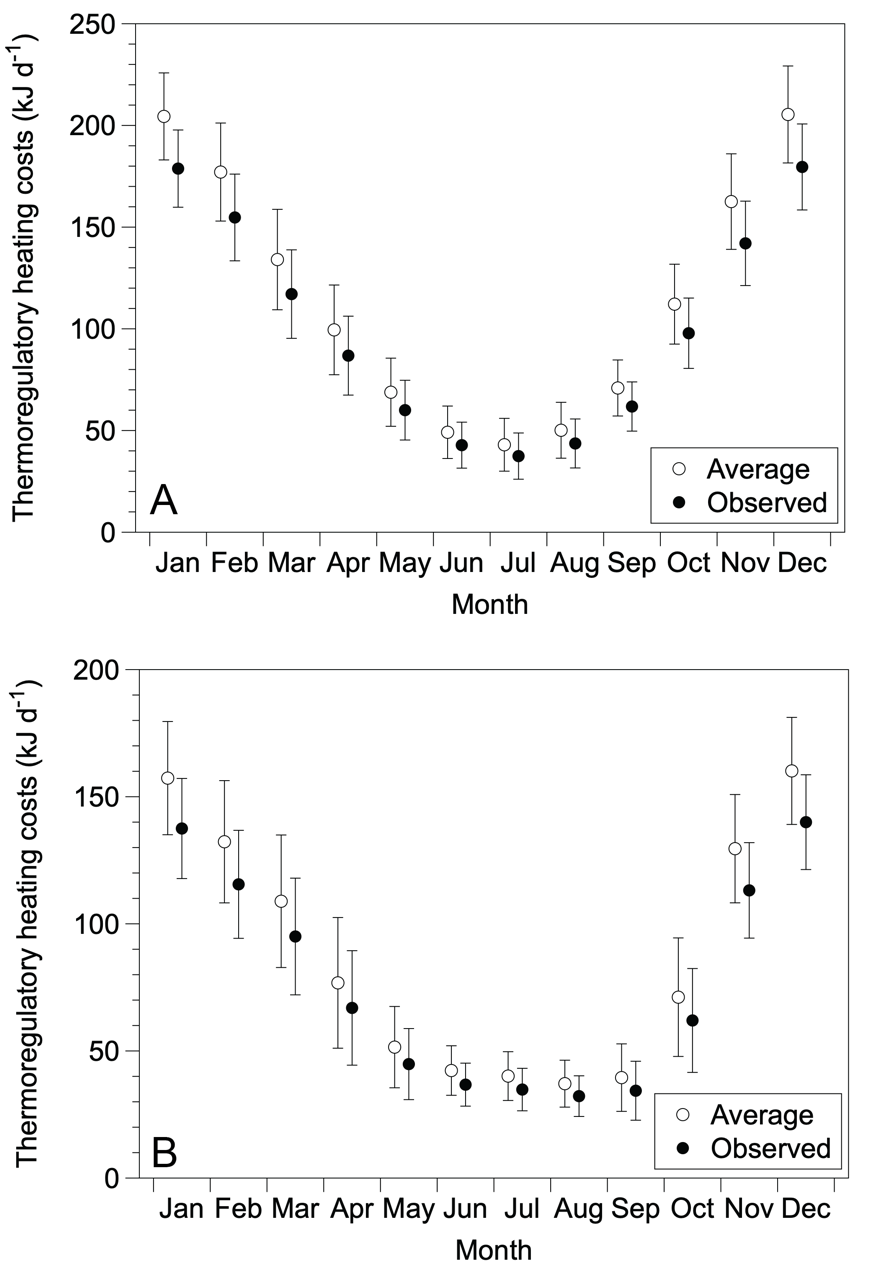
**
